# Real time optical Biopsy: Time-resolved Fluorescence Spectroscopy instrumentation and validation

**DOI:** 10.1038/srep38190

**Published:** 2016-12-08

**Authors:** David S. Kittle, Fartash Vasefi, Chirag G. Patil, Adam Mamelak, Keith L. Black, Pramod V. Butte

**Affiliations:** 1 Cedars Sinai Medical Center, Department of Neurosurgery, Los Angeles, 90048, USA

## Abstract

The Time-resolved fluorescence spectroscopy (TR-FS) has the potential to differentiate tumor and normal tissue in real time during surgical excision. In this manuscript, we describe the design of a novel TR-FS device, along with preliminary data on detection accuracy for fluorophores in a mixture. The instrument is capable of near real-time fluorescence lifetime acquisition in multiple spectral bands and analysis. It is also able to recover fluorescence lifetime with sub-20ps accuracy as validated with individual organic fluorescence dyes and dye mixtures yielding lifetime values for standard fluorescence dyes that closely match with published data. We also show that TR-FS is able to quantify the relative concentration of fluorescence dyes in a mixture by the unmixing of lifetime decays. We show that the TR-FS prototype is able to identify in near-real time the concentrations of dyes in a complex mixture based on previously trained data. As a result, we demonstrate that in complex mixtures of fluorophores, the relative concentration information is encoded in the fluorescence lifetime across multiple spectral bands. We show for the first time the temporal and spectral measurements of a mixture of fluorochromes and the ability to differentiate relative concentrations of each fluorochrome mixture in real time.

Glioma, the most common primary malignant brain tumor, has a very poor prognosis with an average life expectancy of 1–3 years from the time of diagnosis^1^. Near complete surgical removal remains the single most effective treatment to improve quality of life, progression free survival and overall survival^2^,^3^,^4^,^5^. Surgical excision of primary brain tumors is often limited by the surgeon’s ability to differentiate normal brain tissues from tumor, particularly at the margins where these tumors are notoriously infiltrative. Unlike other organs in which “margin free” resection may not be associated with increased morbidity, removal of normal brain tissue can often be associated with significant neurological deficit. Thus, the neurosurgeon must always balance between maximizing extent of resection and avoiding injury to healthy brain structures. Several methods have been employed to resolve this problem, including image guided surgery^6^, and intra-operative MRI^7^, but these approaches have significant drawbacks of either not providing the surgeon with real-time information about the extent of tumor resection while the resection is being performed or disrupting the usual surgical workflow. Similarly, repeated intraoperative frozen section biopsies read by the pathologist are impractical and time consuming.

In the early 80s, Alfano *et al*. introduced an optical biopsy concept for cancer detection using ultraviolet light absorption and fluorescence emissions to differentiate human malignant breast tissue from normal tissue^8^,^9^. Fluorescence spectroscopy, both continuous and time-resolved can provide optical contrast from the morphological changes occurring in diseased tissue where the emission and absorption of light by biological tissues has been studied comprehensively^10^,^11^,^12^,^13^,^14^,^15^. Time-resolved fluorescence spectroscopy offers advantages over using steady state spectral analysis such as fluorescence decay parameter analysis^16^,^17^. The time-resolved fluorescence measurement of fluorescence mixture dyes in solution were first studied in the early 1980s with a streak camera and rise time and decay time of the fluorescence mixtures has been studied leading to Förster resonance energy transfer (FRET) demonstration between donors and acceptor molecules^18^,^19^. Optical technologies for detecting cancerous tissue based on tissue auto fluorescence spectra or lifetime have been investigated^20^,^21^,^22^.

Newer technologies such as steady-state Fluorescence Spectroscopy (FS)^23^,^24^,^25^,^26^,^27^,^28^, Time Resolved Fluorescence Spectroscopy (TR-FS)^29^,^30^,^31^,^32^,^33^, Raman Spectroscopy^34^, and Optical Coherence Tomography (OCT)^35^, are being investigated for their potential to identify brain tumors during surgical removal. Real-time “optical biopsy” using techniques such as fluorescence spectroscopy, diffuse reflectance and Raman spectroscopy analyze the intrinsic properties of the metabolites and proteins in the tissues during surgery, and are a promising method to facilitate surgical decision making and maximize extent of resection while minimizing injury to normal brain. Of these methods, lifetime fluorescence spectroscopy (LTFS) is perhaps the most mature technology. We have previously shown the potential of Time-resolved fluorescence spectroscopy (TR-FS) in distinguishing brain tumor from normal brain tissue in a human clinical trial^33^.

TR-FS is a technique, which uses ultra-short laser pulses to excite the tissue sample and record the corresponding fluorescence intensity decay in multiple spectral bands. The fluorescence decay is a robust measure of tissue characteristics and is not affected by fluorescence intensity fluctuations commonly found in intraoperative data. This provides TR-FS an advantage over other optical techniques, which can be susceptible to blood absorption, measurement distance from the probe, and surgical microscope background light.

In this paper we describe a newer generation TR-FS prototype which is able to record tissue autofluorescence in less than one second and is able to identify different fluorescent compounds in near real-time. The TR-FS system is built to differentiate biological tissues based on their intrinsic fluorescence characteristics, which are derived from multiple proteins and metabolites. These proteins and metabolites include NADH, FAD, lipopigments, Protoporphyrin, glutamate decarboxylase (GAD), Pyridoxamine 5-Phosphate (PMP), and others^36^,^37^. Most of these fluorophores have a fluorescence lifetime between 0.5–4 nanoseconds. Furthermore, The Full Width Half Maximum (FWHM) emission spectra of biological tissue fluorophores are normally in the range of 70–150 nm. However, each of the fluorophores also has distinctive lifetime decays. Our TR-FS system is able to simultaneously detect fluorescence lifetime in six spectral bands (band 1: 390–415 nm, band 2:420–450 nm, band 3: 450–480 nm, band 4: 480–550 nm, band 5: 550–600 nm, band 6: >600), which were chosen based on our previous clinical studies^30^,^31^,^32^,^33^. To assess accuracy and sensitivity, we evaluated the ability of our TR-FS prototype to perform quantitative analysis of fluorescence mixtures with overlapping emission spectra but different fluorescence decay.

The design of our TR-FS system was developed to improve the accuracy, reproducibility, and data integrity of our previous system in clinical settings. We demonstrate that the TR-FS prototype is able to quantify lifetime fluorescence decay with high accuracy by determining the fluorescence lifetime of fluorescence standards as well as organic fluorophores. Lastly, we show the robustness of the technique and its ability to perform quantification analysis of compound fluorescence mixtures independent of their fluorescence intensity.

We used the fluorescence standards Rose Bengal and Rhodamine B, as well as NADH, FAD, Protoporphyrin IX and their mixtures with different relative concentrations.

## Results and Discussion

### Organic fluorescence standard dyes

**Figure 1:**
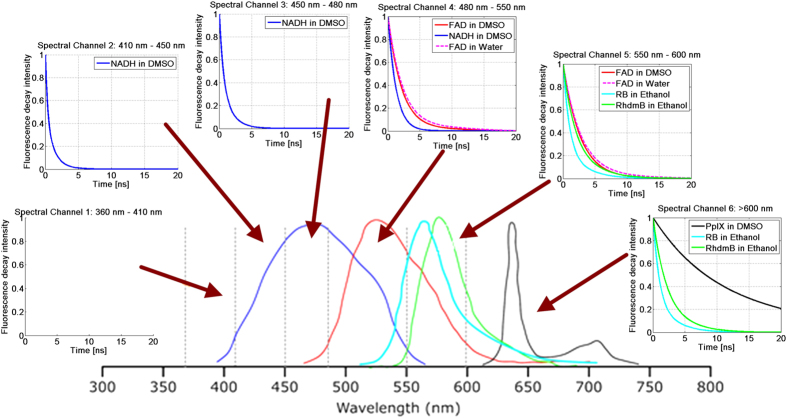
Fluorescence intensity response function of NADH, FAD, PpIX, Rose Bengal, and Rhodamine B compared to their emission spectra.

In order to demonstrate the accuracy and repeatability of the system, a set of well-known organic fluorescence dyes with well-documented fluorescence lifetimes were tested. These include Rhodamine B (RhdmB), Rose Bengal (RB), NADH, FAD and Protoporphyrin IX (PpIX) (Fig. 1). Table 1 shows the lifetime value obtained by the TR-FS prototype from 20 independent measurements, along with the accepted value for this lifetime value from previous publications^38^. As seen in Table 1, the system demonstrates high concordance with accepted lifetime values with a very low (21 ps) standard deviation. Table 1 shows TR-FS measures fluorescence lifetime in six spectral bands from 365 nm to 700 nm (see Fig. 1).

The fluorescence intensity response function (fIRF) is calculated after deconvolution of measured fluorescence decay with the instrument response function (IRF). The average lifetime is calculated by fitting the fluorescence decay to a standard mono-exponential curve. The lifetime value is found by the interpolated time at which the fIRF decays to 1/e of its maximum value. To document reproducibility, we recorded the fluorescence decay from each fluorophore 20 times. The low standard deviation (Give value) demonstrates that the fluorescence decay measured is highly reproducible and consistent. Fluorescence decay from RhdmB and RB solutions is recorded in all six bands. No discernible fluorescence was observed in bands 1–4. RhdmB has fluorescence lifetimes of 2.48 ns (std:0.097) in band 5 and 2.65 ns (std:0.0098) in band 6. RB has fluorescence lifetimes of 770 ps (std:5) in band 5, and 670 ps (std:18) in band 6.

To obtain high accuracy measurement using TR-FS we needed to overcome to main problems caused by jitter and low signal-to-noise measurements. Time resolution due to low digitizer-sampling rate can also lead to sub-sampling error while digitizing a fast signal. The measurement error in digitizing a fast signal can be caused by jitter as well as sub-sampling. Low digitizer-sampling can result in high standard deviations following repeated measurements. For instance, for the dye Rose Bengal lifetime standard deviation is larger than that of Rhodamine B at spectral band above 600 nm of wavelength. The reproducibility of the lifetime estimation also depends on the signal to noise ratio (SNR). This is observed in the measured lifetime of NADH in band 4 (500–550 nm) where the fluorescence emission is low leading to lower SNR. FAD fluorescence emission is red shifted compared to NADH (520–570 nm) with lifetime of 2.9 ns in free form^38^,^39^,^40^. PpIX has emission peak at 635 nm and a secondary peak at 710 nm with a measured lifetime up to 15 ns^38^,^39^,^40^.

By averaging data points, we were able to reduce artifacts caused by both jitter and sub sampling. Additionally, by averaging a signal with random jitter we are equivalently able to super sample the measurements (the measurements are not time-locked). With many measurements, the results of the averaged signals tends toward (due to the repetitive waveform nature of the signal^41^) a super-sampled estimate of the actual signal. The low dynamic range of the digitizer may also restrict our capability to record low intensity fluorescence in one band along with the high intensity in another. This low dynamic range of the digitizer will restrict the range of fluorophores we can measure simultaneously. Based on the fluorescence recorded from human tissue in the previous studies we found the current dynamic range adequate. By averaging 1000 measurements, we can improve signal to noise condition for low intensity fluorescence measurements. However, we are currently attempting to upgrade our digitizers with higher dynamic range and higher bit depth albeit with lower sampling rate.

### Fluorophore mixture

The real-world value of fluorescence lifetime measurements is not simply used to determine the lifetime of a single, mono-exponential dye. In a complex biological system such as tissue, multiple fluorophores combine to give complex, multi-exponential decay curves. In tissue, either the fluorophores may have overlapping fluorescence emission spectra or they may exhibit different concentrations. We demonstrate that by using only the fluorescence lifetime in six spectral bands we are able to qualitatively identify the relative concentrations of the individual fluorophores. As a result, we demonstrate that in complex mixtures of fluorophores, the relative concentrations information is encoded in the fluorescence lifetime across multiple spectral bands. We have shown for the first time the temporal and spectral measurements of a mixture of fluorochromes and the ability to differentiate relative concentrations of each fluorochrome mixture in near real time. Two sets of dyes mixtures were tested for this experimental validation. Organic standard dyes such as RhdmB and RB, were mixed in four different concentrations along with pure RhdmB and RB for reference. The second set includes mixture of organic dyes such as NADH, FAD, and PpIX in DMSO.

**Figure 2:**
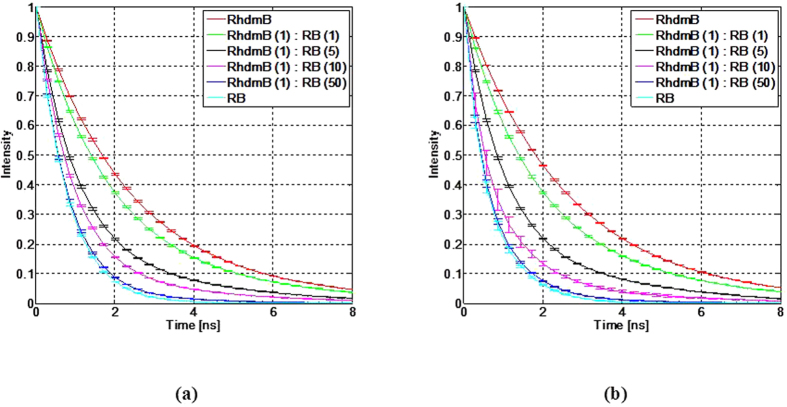
Fluorescence intensity response function of RhdmB and RB mixtures at (**a**) band 5 in 560 nm – 600 nm range, and (**b**) band 6, with wavelengths longer than 600 nm.

The standard fluorescence dyes (RhdmB and RB) were prepared and measured with different volumetric proportions (same concentration), as shown in Fig. 2. Volumetric proportions of RhdmB:RB at 1:1, 1:5, 1:10, and 1:50 were prepared. These concentrations were selected in order to distinguish between lifetimes of similar mixtures since the quantum yield of RhdmB (0.7) is intrinsically higher that that of RB (0.11).

Table 2 shows the average lifetime of RhdmB/RB mixtures obtained from the fIRF. The f*IRF* was calculated after deconvolution of measured fluorescence decay by instrument response function (IRF). As the volumetric proportion of RB increases, the average lifetime decay of the mixture decreases in both spectral bands 5 and 6.

Similarly, mixing NADH, FAD, and PpIX in DMSO will simulate the prominent fluorophores present in a biological tissue environment such as the brain hile the emission spectra expand into all visible wavelengths covering 6 spectral bands in our TR-FS system. As shown in Table 3, NADH[1]:FAD[1] spans more spectral emission bandwidth from band 1 to band 5 in the TR-FS system. The spectral bands where there is no fluorescence emission from FAD are affected less by the presence of FAD. The same scenario is repeated for spectral band 5, where NADH has a very weak fluorescence emission. Spectral band 4 (500–550 nm) has the highest overlap between NADH and FAD emissions, therefore the average lifetime is measured at 1.56 ns, which is in between 1.15 ns and 2.23 ns independently measured at pure NADH and FAD in DMSO (see Table 3). Increasing FAD concentration leads to longer temporal decay as expected in spectral band 5. However, for the lifetime in the spectral range where FAD has very weak emission, the average lifetime decreases slightly. By adding PpIX to the NADH[1]:FAD[1] mixture, not only is emission in spectral band 6 (>600 nm) observed, it also leads to a slight increase in the average lifetime recorded in band 5.

Any individual fluorophore in a given environment has a fluorescence lifetime decay than can be modeled by fitting to an exponential decay function. In some cases, these individual fluorophores are better modeled by fitting to a bi-exponential decay function. To derive parameters from the fluorescence lifetime decay from a complex mixture of fluorophores such as tumor tissue, a more comprehensive view of the decay is required. For tissues with multiple fluorophores, measurements produce complex decay functions that are a combination multiple single or bi-exponential decay function. These complex multi-exponential functions can be composed of fast, normal and slow decay trends. From the decay function (fIRF), we derive seven parameters (τ_0.1_, τ_0.2_, …, τ_0.7_) that are the time values corresponding to 10%, 20%, …, 70% of maximum normalized intensity (fIRF). This gives us reference points to evaluate differences in the slow, medium, and fast components of complex decay functions. The lifetime at each of these points will change differentially and in proportion to mixture concentration.

**Figure 3:**
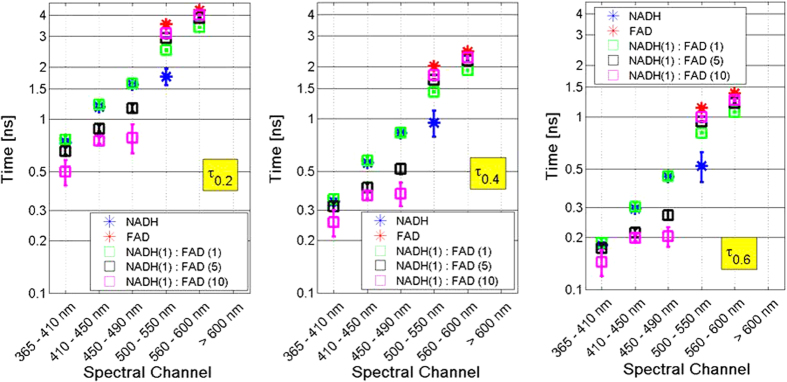
Fluorescence lifetime spectra of NADH and FAD mixtures shows different slow (left), normal (middle) and fast (right) decay lifetimes in six spectral bands.

Figure 3 shows the τ parameters for NADH only, FAD only, and three mixtures with different concentrations. The τ parameters are measured at six different spectral bands. The three box plots show the mean and standard deviation (20 independent measurements) for the τ parameters for each of the spectral bands for τ_0.2_, τ_0.4_, and τ_0.6_. In these plots, τ_0.6_ represents fast decay, τ_0.4_ represents medium decay, and τ_0.2_ represents slow decay components.

Spectral band 4 clearly shows the lifetime differentiation between NADH, FAD and their mixtures, since this is the only spectral band where NADH and FAD emission spectra are overlapping. In this case, we are showing the mixture of two fluorophores however in tissue, we also have contribution from collagen, porphyrins, lipofuscin, flavins and other natural fluorescent constituents. For these more complex mixtures, by relying on information from multiple wavelength measurements, we can disentangle the relative contributions of multiple components by the combination of emission spectra and decay characteristics.

**Figure 4:**
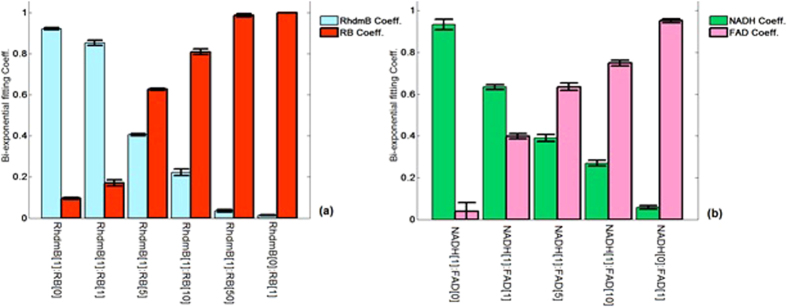
Bi-exponential fitting coefficients in (**a**) RhdmB/RB mixtures, (**b**) NADH/FAD mixtures.

Although previous techniques have utilized fluorescence intensity spectra to un-mix compound fluorescence mixtures, we hypothesize that using only the fluorescence lifetime it is possible to characterize the different tissues. We demonstrate that in complex mixtures of fluorophores, the relative concentrations and quantum yield information is encoded in the fluorescence lifetime across multiple spectral bands. Where the fluorophore samples contain a mixture of fluorophores, it is possible to quantify unknown relative concentrations by analyzing the fluorescence decay of the fluorochrome mixture. For example, we can numerically fit the measured fIRF from a mixture of two fluorochromes (NADH/FAD or RhdmB/RB) to a standard bi-exponential function using the known lifetime values of pure fluorophores and determine the relative concentration of the two fluorophores. Figure 4 shows the two coefficients of bi-exponential fitting parameters in the RhdmB/RB and NADH/FAD mixtures derived from fIRF in spectral bands of 5 and 4 (chosen as the highest emission intensity), respectively. The formula used for the fitting is:


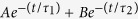


where A and B can be the coefficients between [0, 1] values corresponding to the fluorophores with lifetime values of *τ*_1_ and *τ*_2_, respectively. Each coefficient is dependent on the relative concentration of the dye as well as the fluorophore quantum yields. Figure 4(a) shows the bi-exponential coefficients of RhdmB and RB and their different mixtures. Since the RhdmB quantum yield is more than 6 times greater than that of RB, the RhdmB coefficient is much higher than the RB coefficient in the RhdmB[1]:RB[1] mixture. However, the RhdmB and RB coefficients are closer to 50% in the RhdmB[1]:RB[5] mixture. The error bars represent the standard deviation calculated from 20 independent measurements at each mixture type. Figure 4(b) represents the NADH and FAD coefficients at different mixture concentrations. It is noticed that NADH/FAD shows less quantum yield difference compared to RhdmB/RB. The error bars show that the variability over multiple measurements is small (~ ±2% of sum of the coefficients). This means that the relative fluorescence concentrations can be calculated with reasonable accuracy and reproducibility from the bi-exponential fitting coefficients for the measured mixture fIRF.

### Near real-time data acquisition and Analysis

The current TR-FS prototype is able to estimate the fluorescence lifetime in near-real time and identify the fluorescence mixtures based on fluorescence lifetime parameters. We demonstrate this by training the TR-FS to identify the samples of Rose Bengal and Rhodamine B and their four different mixtures (see the Supplementary Video). The video also demonstrates the repeatability of fluorescence lifetime measurements of each individual sample.

### Tissue fluorescence quantification in cancer diagnostics

We have demonstrated that in the mixtures of NADH/FAD, the relative concentrations information can be extracted from fluorescence decay fitting to the bi-exponential function. This approach is highly desirable since fluorophore lifetimes can be used as a signature of tissue characterization. For example, in brain cancer applications, it has been shown that there is more NADH activity in the cortex, which offers faster decay compared to tissues with lower NADH activity where the fluorescence decay is dominated by FAD^42^,^43^. This hypothesis matches a previous study, which investigated redox ratios (FAD/(NADH + FAD)) in the rat model^42^. The study showed that the redox ratio in tumors is higher compared to normal tissue, confirming that there is less NADH activity in tumors. Using a different technique, recent proteomic analysis of brain tumors showed a higher degree of NADH-UO 24 down-regulation in glioblastoma multiform compared to normal tissue^43^. Therefore, the TR-FS technique can be used as a diagnostic tool to quantify and monitor NADH/FAD changes in biological tissues by using lifetime properties of fluorescent decay that remain unaffected by environmental factors such as blood absorption. A clinical trial evaluating brain tumor and normal brain decay signatures using our TRFS system has been underway at Cedars-Sinai Medical Center. This intraoperative clinical trial aims to establish sensitivity, specificity and positive predictive value of our TRFS system.

## Materials and Methods

### TR-FS Instrumentation

**Figure 5:**
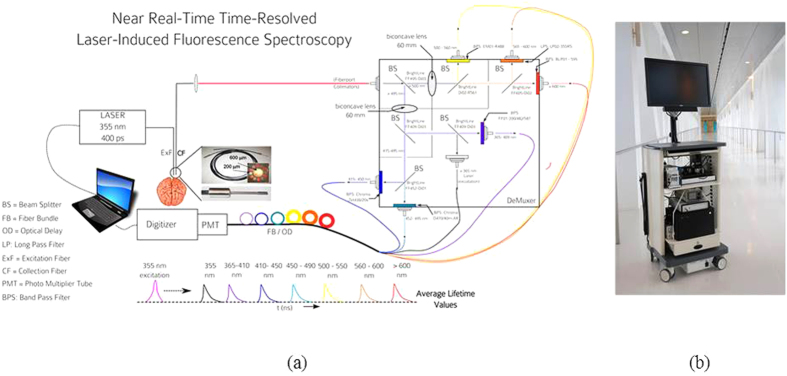
(**a**) Schematic of the TR-FS system, showing the spectral demuxer, optical delay fibers (400 μm 0.22NA), digitizer (7 GS/sec), PMT (80 us rise time) and laser excitation (355 nm, 350 ps pulse). (**b**) The TR-FS system in transit to the operating room at Cedars-Sinai Medical Center. (The brain and laptop artworks source: www.openclipart.org).

The TR-FS system was designed and built for the purpose of intraoperative data collection during brain tumor resection. The subsystems of the instrument are shown in Fig. 5. These components are described in detail in the supplementary information. Briefly, the components consist of a 355 nm short pulse laser running at 1 kHz, an 80 picosecond fast rise-time photomultiplier tube, 7-gigasample-per-second digitizer, spectral demuxer consisting of dichroic beam splitters to isolate six spectral bands from 365 nm to >650 nm, an optical fiber delay to space the six spectral bands in time by 50 ns, and an optical fiber probe with separate excitation and collection fibers to reduce autofluorescence contamination which occurs due to fluorescence from the excitation fiber. This can be a significant issue if the fluorescence from the tissue is weak. The custom probe is sterilizable for safe intraoperative data collection. Each component was secured on a medical grade cart and certified for use in the operating room environment by Underwriters Laboratories. The described system has been used in nearly one hundred surgical procedures at Cedars-Sinai Medical Center (Los Angeles), in an IRB approved clinical trial. Further detailed description of the system hardware is provided in the supplementary information.

### Data Analysis and classification

The fluorescence intensity from TR-FS is modeled as the convolution integral of pure fluorescence decay components from fluorescence material and the optical and electronic components transfer functions known as Instrument Response Function (IRF) since the excitation laser pulse is not a delta-function and the instrumentation additionally has a certain electronic response time. In this paper, we have employed fluorescence dyes with extremely fast decay response to mimic the IRF calculation. More details about IRF measurements are described in the supplementary information. To estimate fluorescence intensity response function fIRF of a sample, the instrument response must be deconvolved from the measured fluorescence intensity. We have employed the “Laguerre expansion of kernels method” to determine the fIRF. The method is based on the expansion of orthonormal set of discrete time Laguerre Functions (LFs). The Laguerre parameter α (0 < α < 1) determines the rate of exponential (asymptotic) decline of the discrete LFs. The optimum selection of the parameter α is important in achieving accurate fIRF estimations. More details on deconvolution steps can be found in our previous work^12^,^13^. In the classification, linear discriminant analysis (LDA) is used as a supervised learning algorithm. The training inputs use selected fluorescence decay lifetime points (e.g. τ[0.1], τ[0.2], …, τ[0.7]) at the six spectral bands with maximized statistical significance difference between training groups.

### Laser Safety Evaluation

The energy output of the laser at the probe tip is 4 μJ, giving the fluence per pulse at the tissue sample of 2 μJ/mm^2^. The current accepted energy of 6.0 mJ/cm^2^ or 60 μJ/mm^2^ by the American Conference of Governmental Industrial Hygienists (ACGIH) is 30 times above this value. At a repetition rate of 1 millisecond, this gives an average power of 2 mW/mm^2^. The maximum permissible exposure (MPE) allowed at UV-A (315–400 nm) is 1 mW/cm^2^ (0.01 mW mm^2^) for 8 hours. Over the short duration of tissue exposure, even though 200 times higher than the MPE, it is well below damage threshold due to low energy of each pulse.

### Experimental Method

We test for accuracy, reliability and data integrity by measuring the fluorescence lifetime of both known organic fluorophores. We demonstrate the accuracy of the system and capability of TR-FS prototype to recover the fluorescence lifetime from dye solutions. The common dyes used are Rhodamine B, Rose Bengal dyes (dissolved in ethanol), NADH, FAD and Protoporphyrin IX (dissolved in DMSO). The measurements demonstrate the accuracy and repeatability of the lifetime decay. Additionally, in order to demonstrate the capability of TR-FS in clinical settings, where fluorescence emission intensity varies based on the presence of blood and/or variation in absorption and scattering properties of tissue, we show that the fluorescence lifetime information is capable of separating small variations in mixtures of fluorophore concentrations while ignoring the variation in the intensity values.

### Fluorescence dye preparation

Rose Bengal (Sigma 330000) and Rhodamine B (Sigma 252425) were prepared in ethanol at 100 μM concentrations. The two fluorescence dyes have similar emission spectra while they have very different fluorescence lifetime and quantum yields. Four mixtures of RhdmB and RB were prepared and measured with different volumetric proportions. RhdmB intrinsically has a higher quantum yield (0.7) compared to RB (0.11) therefore the 1:1, 1:5, 1:10, and 1:50 volumetric proportions for RhdmB:RB have been chosen.

To evaluate the ability of TRFS to differentiate the endogenous fluorescence mixtures mimicking biological tissues, three biomolecules including β-Nicotinamide adenine dinucleotide reduced disodium salt hydrate (NADH) (Sigma N8129), flavin adenine dinucleotide disodium salt dehydrate (FAD) (Sigma F6625), and Protoporphyrin IX (PpIX) (Sigma P8293) were used. The NADH, FAD, and PpIX were dissolved in Dimethyl sulfoxide (DMSO) (Sigma D4540) solutions individually, while PpIX was dissolved only in DMSO. The concentration of NADH, FAD, and PpIX are 1 mM, 0.1 mM, and 0.1 mM, respectively. All samples were measured after they are freshly prepared at room temperature. Four combinations of fluorophore mixtures including NADH[1]:FAD[1], NADH[1]:FAD[5], NADH[1]:FAD[10], NADH[1]:FAD[1]:PpIX[1] all in DMSO were measured and analyzed.

## Additional Information

**How to cite this article**: Kittle, D. S. *et al*. Real time optical Biopsy: Time-resolved Fluorescence Spectroscopy instrumentation and validation. *Sci. Rep.* **6**, 38190; doi: 10.1038/srep38190 (2016).

**Publisher's note:** Springer Nature remains neutral with regard to jurisdictional claims in published maps and institutional affiliations.

## Supplementary Material

Supplementary Information

Supplementary Video

## Figures and Tables

**Table 1:** Standard fluorescence lifetime measured by TR-FS.

Dyes	Solution	Literature	CH1 (365–410 nm)		CH2 (410–450 nm)		CH3 (450–490 nm)		CH4 (500–550 nm)		CH5 (560–600 nm)		CH6 (>600 nm)	
			mean (ns)	std (ps)	mean (ns)	std (ps)	mean (ns)	std (ps)	mean (ns)	std (ps)	mean (ns)	std (ps)	mean (ns)	std (ps)
RhdmB	Ethanol	2.85 ns–2.87 ns									2.48	9.7	2.65	9.8
RB	Ethanol	0.70 ns –0.87 ns									0.77	5	0.67	18
NADH	DMSO	300 ps to 2.0–2.3 ns			0.79	11.8	1.03	20.8	1.15	99.7				
FAD	DMSO	2.9 ns							2.23	9.6	2.63	11.4		
PpIX	DMSO	<15 ns											12.44	5.68

**Table 2:** Rose Bengal and Rhodamine B mixture average lifetimes by TR-FS.

Fluorescence Dyes	CH5 (560–600 nm)		CH6 (>600 nm)	
	mean (ns)	std (ps)	mean (ns)	std (ps)
RhdmB[1]:RB[1]	2.16	13.5	2.20	14.3
RhdmB[1]:RB[5]	1.41	10.1	1.42	10.9
RhdmB[1]:RB[10]	1.11	9.1	0.96	11.5
RhdmB[1]:RB[50]	0.81	9.1	0.72	14

**Table 3:** NADH, FAD, and PpIX mixture measured by TR-FS.

Fluorescence Dyes	CH1 (365–410 nm)		CH2 (410–450 nm)		CH3 (450–490 nm)		CH4 (500–550 nm)		CH5 (560–600 nm)		CH6 (>600 nm)	
	Mean (ns)	Std (ps)	Mean (ns)	Std (ps)	Mean (ns)	Std (ps)	Mean (ns)	Std (ps)	Mean (ns)	Std (ps)	Mean (ns)	Std (ps)
NADH[1]:FAD[1]	0.56	20.6	0.81	16.9	1.05	18.8	1.56	13.2	2.12	11.5		
NADH[1]:FAD[5]	0.484	17.6	0.62	19.7	0.81	28.7	1.85	10.9	2.38	13.9		
NADH[1]:FAD[10]	0.36	49.1	0.54	20.7	0.57	31.9	1.99	14.8	2.48	16.6		
NADH[1]:FAD[1]:PpIX[1]	0.56	41.2	0.76	26.1	1.14	20.2	1.68	17.7	2.77	31	13.14	15
