# Supplementary material for: Real time optical Biopsy: Time-resolved Fluorescence Spectroscopy instrumentation and validation
Source: Sci Rep. 2016 Dec 8;6:38190. doi: 10.1038/srep38190 (PMC5144092; doi:10.1038/srep38190)
Supplement: Supplementary Information [file srep38190-s1.pdf]

Supplementary information

TR-FS Instrumentation

As shown in Figure 1, the TR-FS prototype system comprises an ultra-violet laser as an excitation source (355 nm, Teem Photonics) with a narrow pulse-width of 350 ps, pulsing at 1 KHz. The laser emission is transported to the tissue using a custom trifurcated fiber optic cable (600 micron, 0.12 NA, Fiberguide Industries, Stirling, NJ, USA). The tissue fluorescence is captured by a set of 200 micron (0.22 NA) fibers surrounding the central excitation fiber. These collection fibers are combined in a bundle which transports the fluorescence emission to a custom demuxer separating the fluorescence emission into six color bands. Each of the six color bands are put back into fibers of progressively longer lengths using a set of collimators. These long fibers form a delay unit which delays individual color bands by 50 ft. before ending on a Multichannel Plate Photomultiplier Tube (MCP-PMT) (PMT210, Photek, East Sussex, UK). The signal from the MCP-PMT is digitized at 7 Giga-samples/sec in 8 bit resolution, which is recorded to computer memory for deconvolution. The entire process is controlled using timing and synchronization circuitry (NI-6320, National Instruments, Texas, USA).

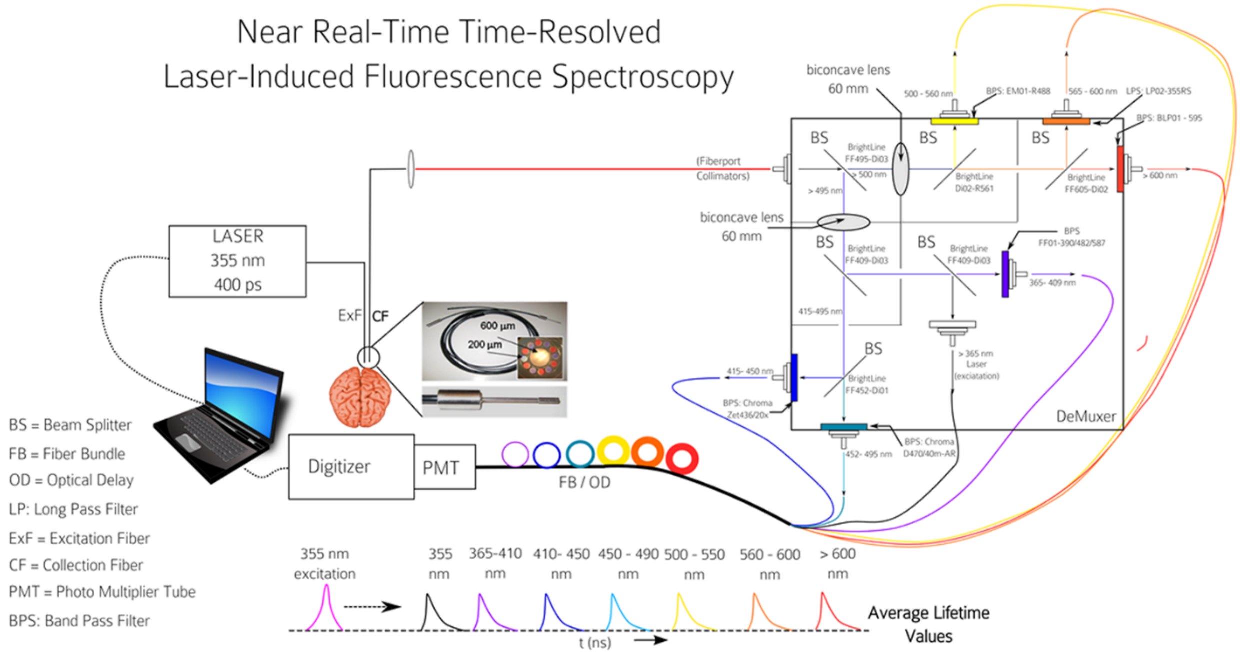

Figure 1 (a) Schematic of the TR-FS system, showing the spectral demuxer, optical delay fibers (400µm 0.22NA), digitizer (7 GS/sec), PMT (80 us rise time) and laser excitation (355nm, 350ps pulse). (b) The TR-FS system in transit to the operating room at Cedars-Sinai Medical Center. The system resides on a medical-grade cart approved for use in hospitals world-wide.

Spectral Demuxer

The spectral channel demuxer (Figure 2) takes the output from six 400 µm 0.22NA collection fibers as a single bundle and separates out the spectral channel through a series of dichroic beam splitters and band-pass filters. The input is collimated by an 11mm focal length aspheric fiber port (PAF-SMA-11-A, Thorlabs) and long-pass filtered at 365nm to block the 355nm laser excitation. The first dichroic beam-splitter reflects the wavelengths shorter than 495nm and transmits longer wavelength of the fluorescence emission. This is the first split with two identical wings, one for the longer wavelengths and one for the shorter to filter the six total bands while minimizing transmission or reflection through more than three dichroic beam-splitters. This configuration also optimizes the dichroic beam-splitters performance since the 495nm split is clean but the 452nm is less efficient and passes through unwanted bands that require band-pass filters for cleanup.

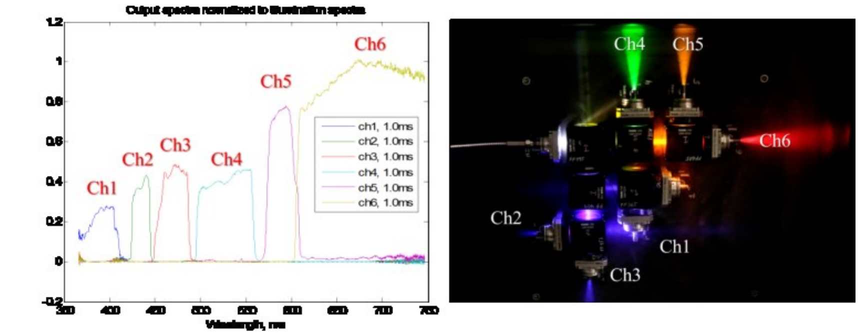

Figure 2: a) Normalized white light source at the input and measured spectral responses of the six channels at the exit ports of the demuxer. The efficiency of the demuxer yields almost 6 times loss without magnification at the output of the fiber ports. (b) Photo of spectral demuxer indicating the bands and spectral throughput of each channel.

TABLE I. Bandpass filters of the spectral demuxer.

Ch. No.	*Filter*	*Bandpass*	*Trans.*
Input [a]	Semrock BLP01-355-25	LP 365nm	>93%
1	Semrock FF01-424/SP-25	350-419nm	>90%
2	Chroma ZET436/20x	427-444nm	>94%
3	Chroma D470/40m-AR	453-488nm	>80%
4	Semrock Em01-R488-25	503-552nm	>90%
5	None	N/A	
6	Semrock	LP 610nm	>93%

[a]Greater than OD 6 blocking for 355nm laser excitation

TABLE II. Dichroic beamsplitters in the spectral demuxer

Filter. No.	*Dichroic BS*	*Edge*	*Refl.*	*Trans.*
1	Semrock FF409-Di03-25x36	409nm	>98% 327-404nm	>93% 415-950nm
1	Semrock FF452-Di01-25x36	452nm	>90% 423-445nm	>90% 460-610nm
2	Semrock FF495-Di03-25x36	495nm	>98% 350-488nm	>93% 502-950nm
3	Semrock Di02-R561-25x36	573nm	>94% 554-568nm	>93% 578-1200nm
6	Semrock FF605-Di02-25x36	605nm	>98% 350-596nm	>93% 612-950nm

[a]Footnote 1

There are two 60mm convex achromat lenses (Thorlabs AC254-060-A-ML) to narrow the divergence of the rays from the fiber ports due to the long distance required between entry and exit fiber ports. Figure 2 also shows relative spectral intensities measured for each of the six channels. Note that the channels with the weakest fluorescence (channels 5 and 6) are matched to the demuxer channels with the highest efficiency.

Optical Delay

A delay line was fabricated with 9 channels ranging in length from 5 ft. to 255 ft. at 50-foot intervals. The six channels on the demuxer are connected to the 5ft, 55ft, 105ft, 155ft, 205 ft., and 255 ft. delay lines. The 50 ft. separation enables measuring longer lifetimes (45 ns total extinction) since for many tissues, the lifetime at 1/e is under 2 ns but the 10% extinction can be quite long. The delay fibers are 400 µm 0.22NA UV grade fused silica fibers and the fabrication of the delay unit was from Fiberguide Industries (Caldwell, ID, USA).

Fluorescent excitation laser source

The laser source is a Teem Photonics (Meylan Cedex, France) PNV-M02510-1x0 passively Q-switched MicroChip laser with 350ps pulse, 1 kHz repetition rate and 25µJ pulse energy output per pulse. There is a photodiode output for synchronization, but the rise time is too slow and the output voltage is too high to trigger the digitizer. Therefore a dichroic beam splitter (Semrock, 365nm long pass) that allows 94% refection and 6% transmission is used to power a separate photodiode (SV2-FC, 2GHz 320-1100nm, Thorlabs). The splitter is directly connected to the laser output with a fiber port to focus the 6% transmission of the beam splitter into a 200µm fiber which in turn connects to the photodiode. The output of the photodiode is terminated into the 50 Ohm trigger input on the digitizer.

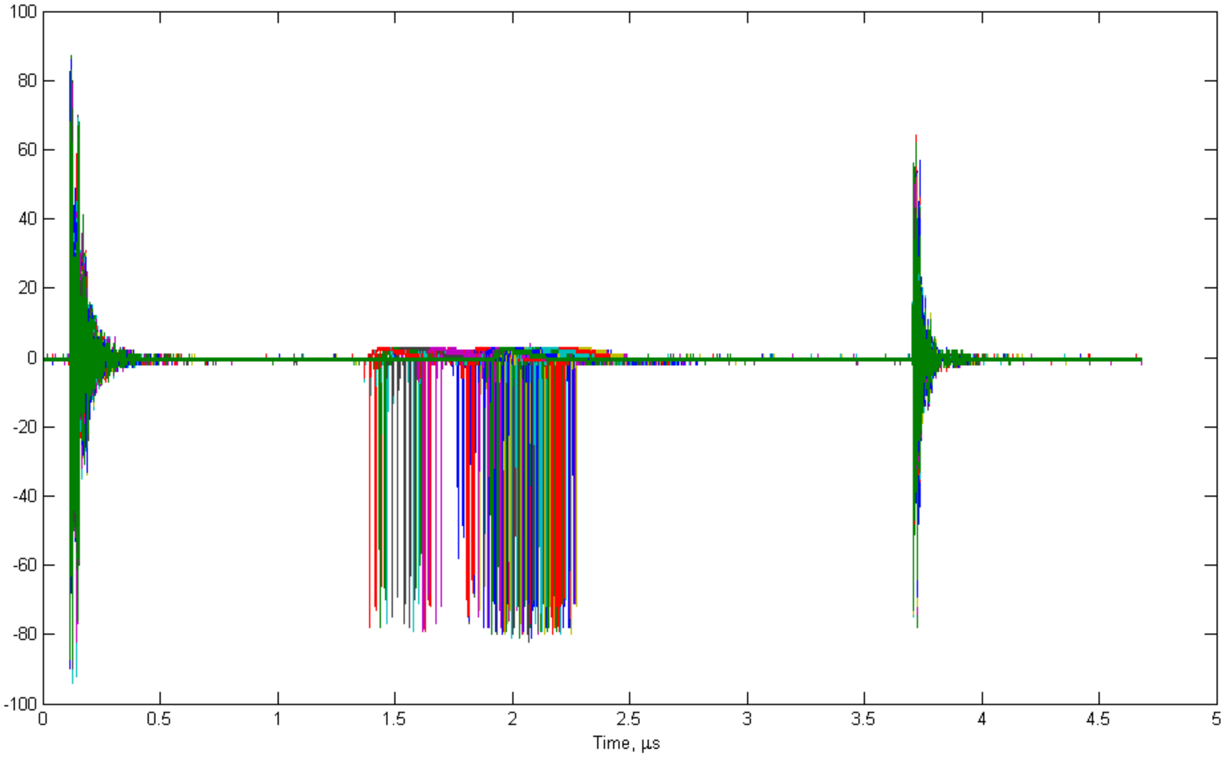

Figure 3 PMT output captured by the digitizer showing laser pulse jitter and settling time of the PMT and gate module. The window that the PMT is one must account for this jitter and settling time to ensure complete capture of the fluorescence signal.

Collection and Excitation Probe

The excitation laser pulse is transmitted to the sample by a 3.0 meter 0.11NA 600µm UV-grade silica non-solarizing multimode, step index fiber (probe manufactured by FiberGuide) and the collection is carried by twelve 0.22NA 200µm fibers surrounding the excitation fiber (Figure 4). The collection fibers are beveled at a 10-degree angle in order to improve excitation collection overlap for small distances between the probe tip and sample. The collection fibers are located on a concentric circle 480 microns in radius around the central excitation fiber. These twelve fibers are then divided into two bundles of six each for two subminiature version A (SMA) terminated, bundled collection fibers. Every other fiber is routed to the opposing collection fiber. The fibers are built into a rigid, metal probe 7-cm long and at the end of the 3.0 meters of flexible cable. The entire probe is Sterrad (Steris) sterilizable up to fifty times without significant loss in transmission. Separate collection and excitation fibers are used to minimize the intrinsic fluorescence of the fiber due to the high-power of the excitation laser. The optimal distance for the probe tip is 3 mm above the tissue sample giving an illumination area defined by the NA of the excitation fiber of 2 mm^2.

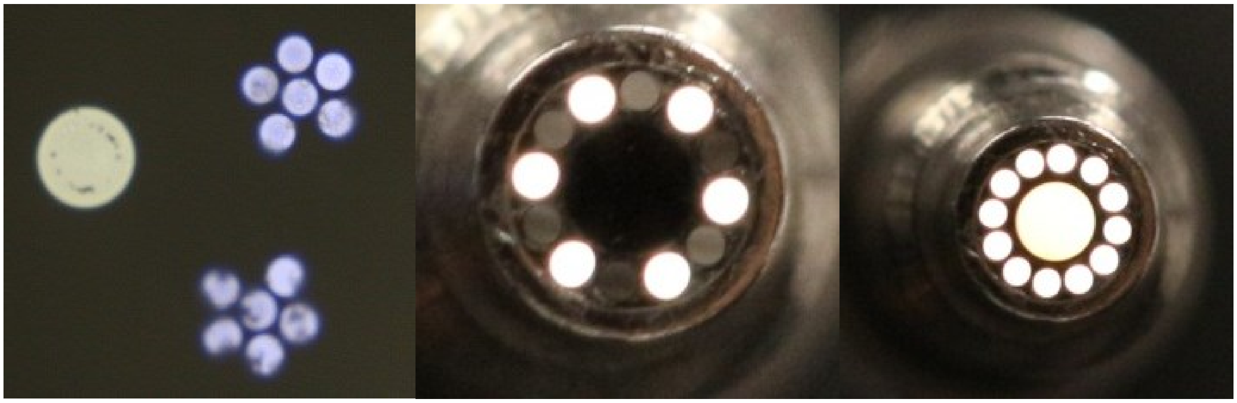

(a) (b) (c)

Figure 4 Photographs of the collection and excitation fibers on the probe. (a) image of the collection and excitation fibers on a sheet of paper, showing some damage to the fibers due to sterilization. (b) six collection fibers for a single bundle for input into the demuxer. (c) both collection fibers and excitation fiber illuminated, showing the configuration of the probe head end.

Laser Safety

The energy output of the laser at the probe tip is 4 μJ, giving the fluence per pulse at the tissue sample of 2μJ/mm^2. The current accepted energy of 6.0 mJ/cm^2 or 60 μJ/mm^2 by the American Conference of Governmental Industrial Hygienists (ACGIH) is 30 times above this value. At a repetition rate of 1 millisecond, this gives an average power of 2 mW/mm^2. The maximum permissible exposure (MPE) allowed at UV-A (315-400nm) is 1mW /cm^2 (0.01mW mm^2) for 8 hours. Over the short duration of tissue exposure, even though 200 times higher than the MPE, is well below damage threshold due to the short duration of pulses and low energy of each pulse.

Digitizer and photomultiplier tube

The output of the spectral demuxer is mixed and collected by a Photek photomultiplier tube (PMT), model PMT210 with an 80 ps rise time and gain of 1 million. The PMT is gated for 10 μs for each laser pulse due to a 2-3 μs jitter of the laser pulse and a 500ns settling time of the gate module (Photek GM200-3N). The PMT is supplied by a remotely controlled high voltage supply (Photek) that allows for automatic adjustment of the PMT voltage for various fluorescence signal strengths.

The PMT voltage ranges between -3 kV and -4.2 kV with a maximum permissible value of -5 kV. Synthetic dyes that are highly fluorescent require a voltage of -3.0 kV to -3.4 kV. Tissue samples can require up to 4.2 kV, especially in the operating environment where the probe tip can get dirty. To accommodate this, after each pulse sequence, the signal intensity from the digitizer is assessed for saturation or low signal and a corresponding look-up table is accessed to set a new voltage for the PMT that optimizes the collection sensitivity. Due to the ramp-up of the PMT voltage, this usually takes a few cycles before stabilizing to the set voltage.

The output of the PMT is digitized by a digitizer (SP-Devices ADQ108) at 7 Giga-samples per second with 8-bit resolution. The output of the fiber delay yields a 120-foot separation between the first channel in time and the last channel or 382.5 ns. Fluorescence decay in each band is measured for 22.5 ns, so the total time that the digitizer measures for each laser pulse is 202.5 ns or 1400 samples. Currently, the digitizer captures 2048 samples per pulse with the first pulse occurring at sample 150 and the sixth pulse peak occurring at sample 1400. The additional 600 data points are used to calculate the offset preference for the digitizer after the signals from multiple laser pulses have been averaged. This gives a non-biased signal for lifetime calculation. Depending on signal integrity and strength, 10-100 pulses are averaged and combined with each of the laser pulses separated by 1 millisecond. For 100 pulses, acquisition, download, and lifetime processing occurs in 200ms. For stronger fluorescent signals requiring only 10 laser pulses, acquisition runs at near real-time in 40-60ms. Biological sources tend to show high variability in the raw fluorescence pulses, yielding higher standard deviations in lifetime measurements. For some tissues, up to 1000 measurements are required for good repeatability. Therefore, all clinical data is captured using 1000 averaged laser pulses.

Calibration and standardization

The fluorescence decay measured at the digitizer is broadened by multiple factors: 1) finite pulse width of the laser (350ps), 2) finite bandwidth of the PMT and digitizer electronics, 3) intermodal dispersion in the multimode fibers used for the probe collection and excitation and the delay fibers. The actual impulse response of the system at each spectral band is then required to recover the fluorescence decay measured by the system since the measured signal is the convolution of the instrument response function and laser pulse width.

Data Processing and Deconvolution

The intrinsic fluorescence impulse response functions for each spectral channel can be recovered by numerical deconvolution of the measured system impulse response. Given a characterization of a linear time-invariant (LTI) system, the input x(t) is mapped to the output y(t) through the system H. In context of TRLIFS, the system obeys both superposition (the output is a linear combination of any input signals) and the system is time-invariant (adding delay to the input signal only adds delay to the output) and can be modeled by a Laguerre expansion of kernels for deconvolution to directly recover the system models.

The Laguere functions $b_j^a(n)$, which are orthonormal, are used to expand the impulse response functions and estimate the Laguerre expansion coefficients c_f. The fluorescence impulse response functions are then estimated for each of the six spectral channels and the steady-state spectrum can then be computed by integrating each intensity decay curve with respect to time.

Instrument Response function

In order to derive the actual fluorescence lifetime it is important to eliminate the effect of the optical and electronic components. This is achieved by deconvolving the instrument response. We have suggested employing fluorescence dyes with extremely fast decay response to mimic the IRF calculation. We chose 4-dimethylamino-4-cyanostilbene (DCS, ChemBridge Corporation, San Diego, CA) solution in Cyclohexane, 25°C, with emission range of 300 nm -500 nm to cover IRF for spectral channels 1-4. For channel 5 and 6, we used 1 mM concentration of the 2-(p-dimethylaminosotyryl)pyridylmethyl iodide (2-DASPI, Sigma- Aldrich Cat. 280135) in ethanol with maximum emission spectra at 550 nm. The DCS and 2-DASPI average lifetime is 66 ps and 30 ps, respectively.

In order to calculate pure fluorescence intensity decay from the TR-FS measurement, we need to independently characterize the instrument response function (IRF). The IRF can be determined by evaluation of the optical throughput including multimode fiber dispersion in probe and delay lines, laser optical properties, spectral demuxer, and detector

Lifetime Measurement Validation

Accurate lifetimes over all six spectral channels depend on an accurate system response function measurement and proper data fitting during deconvolution, which includes choosing the alpha decay constant and order of the Lagurre Polynomial. The decay constant alpha has a significant effect on lifetime calculation, shown in Figure 5. The order likewise effects lifetime, shown in Figure 6. The dye used for this comparison is K4-503-FLT (SETA BioMedicals) with a fluorescence peak at 415nm and lifetime of 1.25ns. Figure 6 also demonstrates the dependence of wavelength on impulse response and deconvolution. The impulse response for all three channels was measured at 355nm rather than the correct wavelengths, effectively shortening the lifetime calculation with longer wavelength channels.

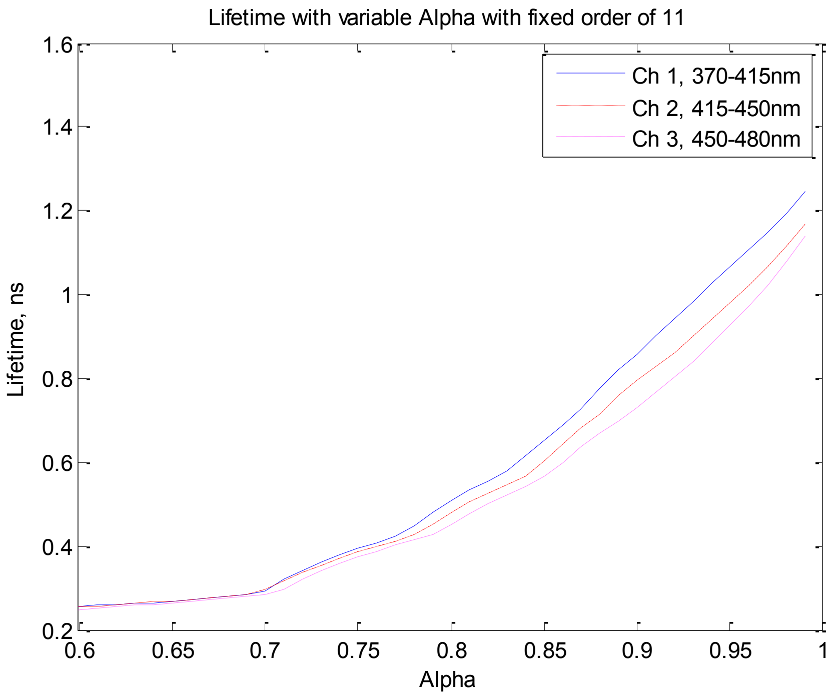

Figure 5 Alpha versus lifetime (fixed order at 11)

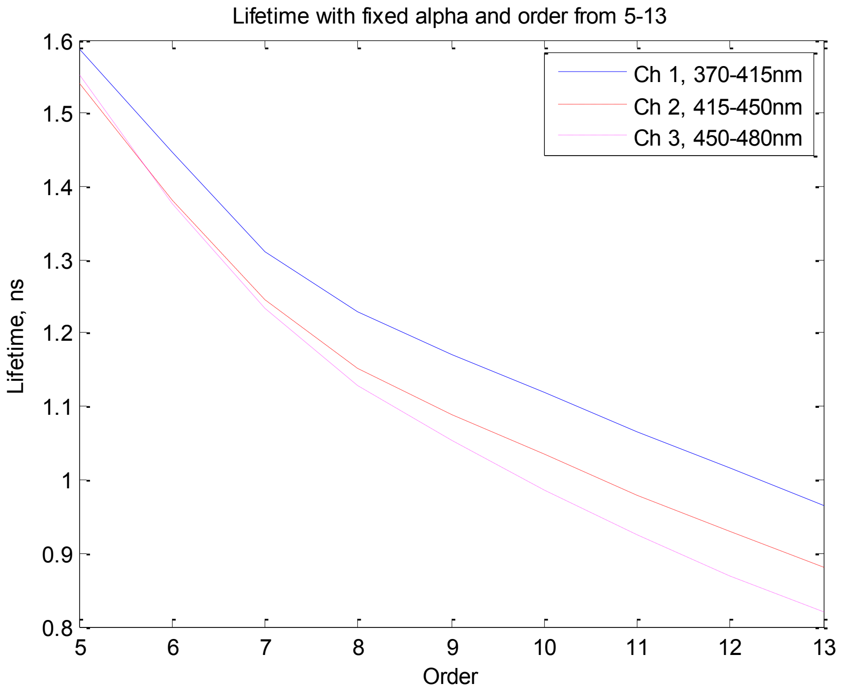

Figure 6 Order versus lifetime (fixed alpha at 0.95) showing the dependence of choosing the correct values for deconvolution. The dye is K4-503 with a 1.25ns lifetime from 360-475nm. An order between 7-9 and alpha of 0.9-0.96 yields the correct lifetime for this dye

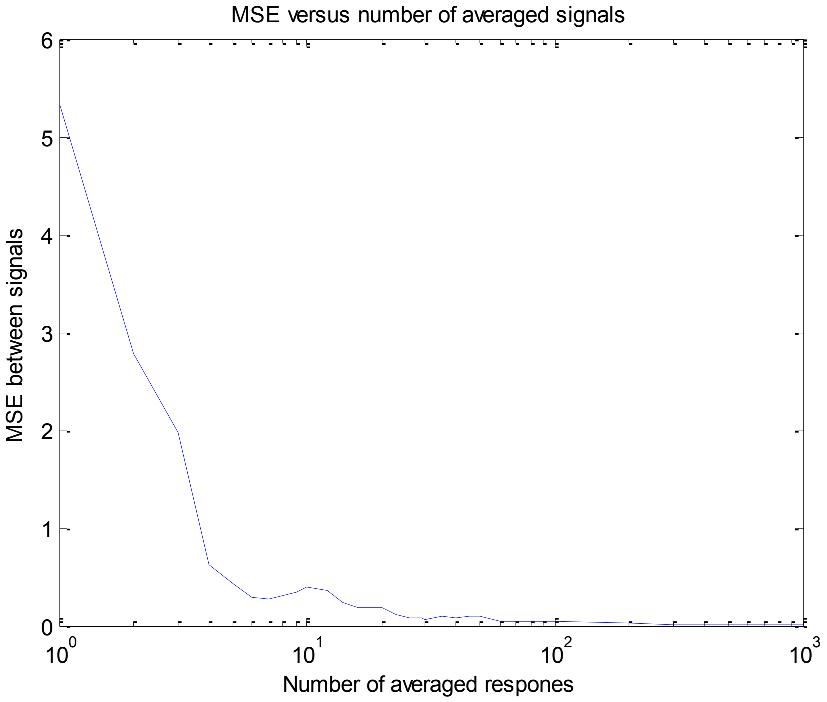

Figure 7 MSE versus number of averaged signals comparing the signal variation between acquisitions. Note that after 30 averages, the MSE drops very little, showing little reduction in error, the signals are almost on top of each other and by 300 averages, there is almost no distinction between them. The error continues to drop beyond 300, but little value is gained in the additional measurements. For weaker signals, this trade-off can increase even more, therefore 1000 averages are always acquired for future flexibility.

Supplementary video legend: The supplementary video shows the TR-FS prototype which calculates the fluorescence lifetime in near-real time and classifies the fluorescence mixtures based on fluorescence lifetime parameters. We demonstrate this by training the TR-FS to identify the samples of Rose Bengal and Rhodamine B and their four different mixtures. The scatterplot shows the classification marker which moves to the predefined regions (defined during the training procedure) while the optical probe moves between each fluorescent mixture.
